# Serum Autotaxin Is a Useful Disease Progression Marker in Patients with Primary Biliary Cholangitis

**DOI:** 10.1038/s41598-018-26531-0

**Published:** 2018-05-25

**Authors:** Satoru Joshita, Takeji Umemura, Yoko Usami, Yuki Yamashita, Gary L. Norman, Ayumi Sugiura, Tomoo Yamazaki, Naoyuki Fujimori, Takefumi Kimura, Akihiro Matsumoto, Koji Igarashi, Kaname Yoshizawa, Masao Ota, Eiji Tanaka

**Affiliations:** 1 Department of Medicine, Division of Gastroenterology and Hepatology, Shinshu University School of Medicine, 3-1-1 Asahi, Matsumoto, 390-8621 Japan; 2 Research Center for Next Generation Medicine, Shinshu University, 3-1-1 Asahi, Matsumoto, 390-8621 Japan; 3 Department of Laboratory Medicine, Shinshu University Hospital, 3-1-1 Asahi, Matsumoto, 390-8621 Japan; 4 Inova Diagnostics, 9900 Old Grove Road, San Diego, CA 92131 USA; 5 Bioscience Division, TOSOH Corporation, 2743-1 Hayakawa, Ayase, 252-1123 Japan; 6 Department of Gastroenterology, National Hospital Organization, Shinshu Ueda Medical Center, 1-27-21 Midorigaoka, Ueda, 386-8610 Japan

## Abstract

Autotaxin (ATX) is a secreted enzyme metabolized by liver sinusoidal endothelial cells that has been associated with liver fibrosis. We evaluated serum ATX values in 128 treatment-naïve, histologically assessed primary biliary cholangitis (PBC) patients and 80 healthy controls for comparisons of clinical parameters in a case-control study. The median ATX concentrations in controls and PBC patients of Nakanuma’s stage I, II, III, and IV were 0.70, 0.80, 0.87, 1.03, and 1.70 mg/L, respectively, which increased significantly with disease stage (r = 0.53, P < 0.0001) as confirmed by Scheuer’s classification (r = 0.43, P < 0.0001). ATX correlated with *Wisteria floribunda* agglutinin-positive Mac-2 binding protein (M2BPGi) (r = 0.51, P < 0.0001) and fibrosis index based on four factors (FIB-4) index (r = 0.51, P < 0.0001). While ALP and M2BPGi levels had decreased significantly (both P < 0.001) by 12 months of ursodeoxycholic acid treatment, ATX had not (0.95 to 0.96 mg/L) (P = 0.07). We observed in a longitudinal study that ATX increased significantly (P < 0.00001) over 18 years in an independent group of 29 patients. Patients succumbing to disease-related death showed a significantly higher ATX increase rate (0.05 mg/L/year) than did survivors (0.02 mg/L/year) (P < 0.01). ATX therefore appears useful for assessing disease stage and prognosis in PBC.

## Introduction

Primary biliary cholangitis (PBC) is a liver-specific autoimmune disease characterized by a female preponderance and the destruction of intrahepatic bile ducts that often causes cirrhosis and hepatic failure^1,2^. The precise etiology of PBC remains unknown but is related to genetic susceptibility, as supported by SNP-based studies^3,4^ and genome-wide association studies across multiple ethnicities^5–10^, as well as environmental factors^1,11^. The major manifestations of symptomatic PBC include fatigue, pruritus, and jaundice. However, the number of patients with asymptomatic PBC is on the rise due mainly to increased awareness and diagnosis at earlier stages by disease-specific antimitochondrial antibodies (AMAs)^12^.

As recommended by most guidelines^13–15^, ursodeoxycholic acid (UDCA) is currently the most effective treatment for PBC and has remarkably improved disease prognosis. Nonetheless, some patients experience progression to cirrhosis, hepatic failure, and rarely hepatocellular carcinoma (HCC)^16,17^. Several factors have been associated with a worsened prognosis, including the presence of symptoms at diagnosis, UDCA unresponsiveness^18^, more advanced histologic stage, presence of antinuclear antibodies^19^, and certain genetic polymorphisms^20,21^. Thus, it is important to accurately diagnose the clinical stage of PBC. Liver biopsy provides essential information regarding the severity of necro-inflammatory activity and liver fibrosis, but is often limited by invasiveness, pain, sampling error, and inter-observer disparity^22^. Simple and reliable non-invasive methods to estimate liver fibrosis^23^ and PBC progression are therefore needed, such as *Wisteria floribunda* agglutinin-positive Mac-2 binding protein (M2BPGi)^24–26^, hyaluronic acid (HA), type IV collagen 7S, aspartate aminotransferase (AST)-to-platelet ratio index (APRI)^16,27^, and fibrosis index based on four factors (FIB-4) index^28^. However, the diagnostic abilities of these markers remain under scrutiny due to their significant, but moderate, accuracy.

Autotaxin (ATX) is a 125 kD type II ectonucleotide pyrophosphatase/phosphodiesterase that was originally isolated as a potent cell motility-stimulating factor from the conditioned medium of A2058 human melanoma cells^29–31^. Nakagawa *et al*. described that lysophosphatidic acid (LPA) and ATX were pathophysiologically involved in liver fibrosis based on the evidence that LPA stimulated proliferation and contractility in hepatic stellate cells^32^. Thereafter, serum ATX has been reported as a novel marker candidate to assess liver fibrosis, histological activity grade, and disease outcome^32–39^. Although the molecular mechanisms involved in the pathogenesis of cholestatic pruritus remain unknown, the ATX-LPA signaling axis is suspected to play an important role based on evidence of increased ATX activity in affected patients^40,41^.

The present study evaluated the performance of serum ATX in predicting histological disease stage in PBC in comparison with currently established indices. We also examined the clinical characteristics of ATX over time in patients with PBC in a longitudinal study.

## Results

### Case-control study for evaluating associations of ATX with disease stage

#### Baseline clinical characteristics

The baseline clinical characteristics in the case-control study to assess the ability of ATX in estimating disease progression are presented in Table 1. Of the 128 enrolled patients, 108 were female and 20 were male. Median age was 57 years. Only 14 patients exhibited symptoms such as variceal bleeding, jaundice, fatigue, and/or pruritus on presentation, with the majority of the cohort being asymptomatic after liver dysfunction was identified as a complication of another disease or following a routine health check-up. In total, 108 of 128 patients (84%) were AMA-positive, 87 of 128 (68%) were ANA-positive, and 32 of 128 (25%) were gp210-positive at the time of diagnosis and liver biopsy. Median nuclear pore glycoprotein p62 (NUP62) value was 2.7 ng/mL. Based on histological findings, the number of patients with Nakanuma’s stage^42^ 1, 2, 3, and 4 was 10, 73, 27, and 18, respectively, and those with Scheuer’s stage^43^ I, II, III, and IV were 84, 18, 15, and 11, respectively. There were significant differences for GGTP (P = 0.002), ALT (P = 0.014), and the fibrosis markers ATX (P = 0.005) and FIB-4 index (P = 0.004) between female and male patients (Table 1).

**Table 1:** Baseline clinical characteristics of 128 patients with primary biliary cholangitis.

Baseline characteristic	Overall (n = 128)		Female (n = 108)		Male (n = 20)		Male vs. female
	Median	IQR	Median	IQR	Median	IQR	P value
Age (years)	57	(51–65)	57	(53–65)	57	(47–68)	0.82
Female/Male	108/20						
Symptoms* at diagnosis	14 (11%)		10 (9.3%)		4 (20.0%)		0.16
**Laboratory data**							
alkaline phosphatase (U/L)	459	(326–605)	445	(322–576)	524	(328–676)	0.48
gamma-glutamyl transpeptidase (U/L)	128	(77–236)	114	(73–210)	288	(149–702)	0.002
alanine aminotransferase (U/L)	40	(27–67)	38	(26–60)	70	(29–103)	0.014
aspartate aminotransferase (U/L)	40	(30–59)	39	(30–59)	50	(34–65)	0.20
total bilirubin (mg/dL)	0.78	(0.59–1.00)	0.76	(0.57–1.00)	0.82	(0.60–1.00)	0.55
albumin (g/dL)	4.2	(4.0–4.5)	4.2	(4.0–4.5)	4.3	(3.9–4.4)	0.51
platelet count (x10^4^/µL)	22.6	(18.3–25.6)	23.0	(18.4–25.7)	21.8	(18.1–25.1)	0.49
immunoglobulin M (mg/dL)	304	(186–518)	296	(171–518)	337	(224–524)	0.51
immunoglobulin G (mg/dL)	1,597	(1,367–1,890)	1,580	(1,367–1,865)	1,650	(1,330–1,963)	0.99
AMA-M2 positive (%)	108 (84%)		90 (83%)		18 (90%)		0.45
anti-nuclear antibody positive (%) (x80)	87 (68%)		73 (69%)		14 (70%)		0.92
anti-gp210 antibody positive (%)	32 (25%)		26 (24%)		6 (30%)		0.57
nuclear pore glycoprotein p62 (ng/mL)	2.7	(1.3–6.5)	2.7	(1.2–6.6)	2.6	(1.5–5.8)	0.69
**Fibrosis markers**							
ATX (mg/L)	0.97	(0.79–1.11)	1.00	(0.82–1.13)	0.78	(0.66–0.98)	0.005
M2BPGi (COI)	0.82	(0.49–1.33)	0.81	(0.47–1.31)	0.89	(0.54–1.62)	0.42
fibrosis index based on four factors index	1.64	(1.21–2.31)	1.75	(1.29–2.39)	1.19	(0.93–1.63)	0.004
aspartate aminotransferase-to-platelet ratio index	0.82	(0.47–0.98)	0.71	(0.47–0.98)	0.82	(0.57–1.00)	0.41
**Histopathological findings**							
Nakanuma’s stage^42^ 1/2/3/4	10/73/27/18		10/61/24/13		0/12/3/5		0.44
Scheuer’s stage^43^ I/II/II/IV	84/18/15/11		73/15/13/7		11/3/2/4		0.33

Data are expressed as the number (%) or median (first-third quartiles). *Symptoms include variceal bleeding, jaundice, fatigue, and pruritus.

Abbreviations: IQR, interquartile range; AMA-M2, anti-mitochondrial antibody specific for the pyruvate dehydrogenase complex-E2 component; ATX, autotaxin; M2BPGi, *Wisteria floribunda* agglutinin-positive Mac-2 binding protein; COI, cutoff index.

#### ATX values and autoantibodies

We compared ATX levels with such disease-specific autoantibodies as AMA-M2 positivity, ANA positivity, gp210 positivity, and NUP62 levels. Median ATX levels were not statistically significant between the positive and negative groups of AMA-M2 (0.97 vs. 0.94 mg/L, P = 0.898), ANA (0.98 vs. 0.96 mg/L, P = 0.346), or gp210 (0.97 vs. 0.97 mg/L, P = 0.982), nor were they correlated with median NUP62 titer (r = 0.00, P = 0.985) (Supplementary Table 1).

#### ATX values in PBC

**Figure 1:**
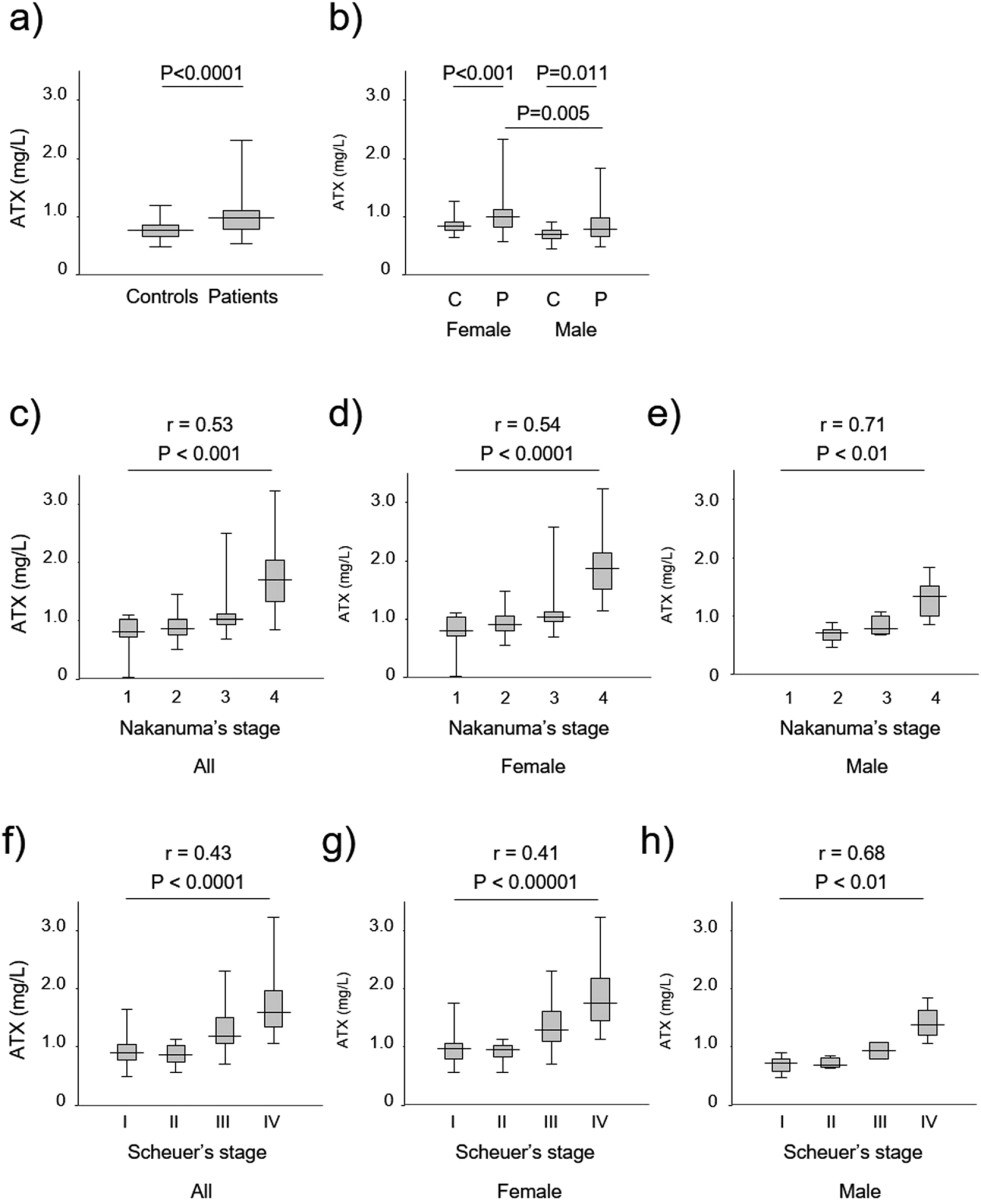
(**a**) Comparison of ATX levels between healthy controls and patients with primary biliary cholangitis. (**b**) Comparison of ATX levels in test groups according to gender. (**c**–**e**) Correlation of ATX and disease stage based on Nakanuma’s classification (**c**) in all patients, (**d**) in females only, and (**e**) in males only. (**f**–**h**) Correlation of ATX and disease stage based on Scheuer’s classification (**f**) in all patients, (**g**) in females only, and (**h**) in males only. The top and bottom of each box represent the first and third quartiles, respectively. The lines across the boxes indicate median values. Abbreviations: autotaxin, ATX; C, controls; P, patients.

The ATX levels of patients with PBC (median: 0.97 mg/L) were significantly higher than those of controls (median: 0.76 mg/L) (P < 0.0001) (Fig. 1a). ATX values in female patients (median: 1.00 mg/L) were significantly higher than those in female controls (median: 0.82 mg/L) (P < 0.001)^36^ as well as in male patients (median: 0.78 mg/L) (P = 0.005). ATX values in male patients (median: 0.78 mg/L) were significantly higher than those in male controls (median: 0.76 mg/L) (P = 0.011) (Fig. 1b).

### Association between ATX values and disease stage according to Nakanuma’s classification

The median ATX values for Nakanuma’s classification stage 1, 2, 3, and 4 were 0.80, 0.87, 1.03, and 1.70 mg/L, respectively, in overall patients^42^. Significant correlations between ATX values and disease progression defined by Nakanuma’s classification were found in overall, female, and male patient groups (overall: r = 0.53, P < 0.0001; female: r = 0.54, P < 0.0001; and male: r = 0.71, P < 0.01) (Fig. 1c–e).

### Association between ATX values and disease stage according to Scheuer’s classification

We compared ATX values with Scheuer’s classification grading to confirm the above associations between ATX and disease stage^43^. The median ATX values for Scheuer’s classification stage I, II, III, and IV were 0.89, 0.86, 1.19, and 1.60 mg/L, respectively, in overall patients. We also observed significant correlations between ATX values and disease progression defined by Scheuer’s classification in overall, female, and male patient groups (overall: r = 0.43, P < 0.0001; female: r = 0.41, P < 0.00001; and male: r = 0.68, P < 0.01) (Fig. 1f–h).

### Correlation of ATX with other clinical and non-invasive fibrosis markers

**Figure 2:**
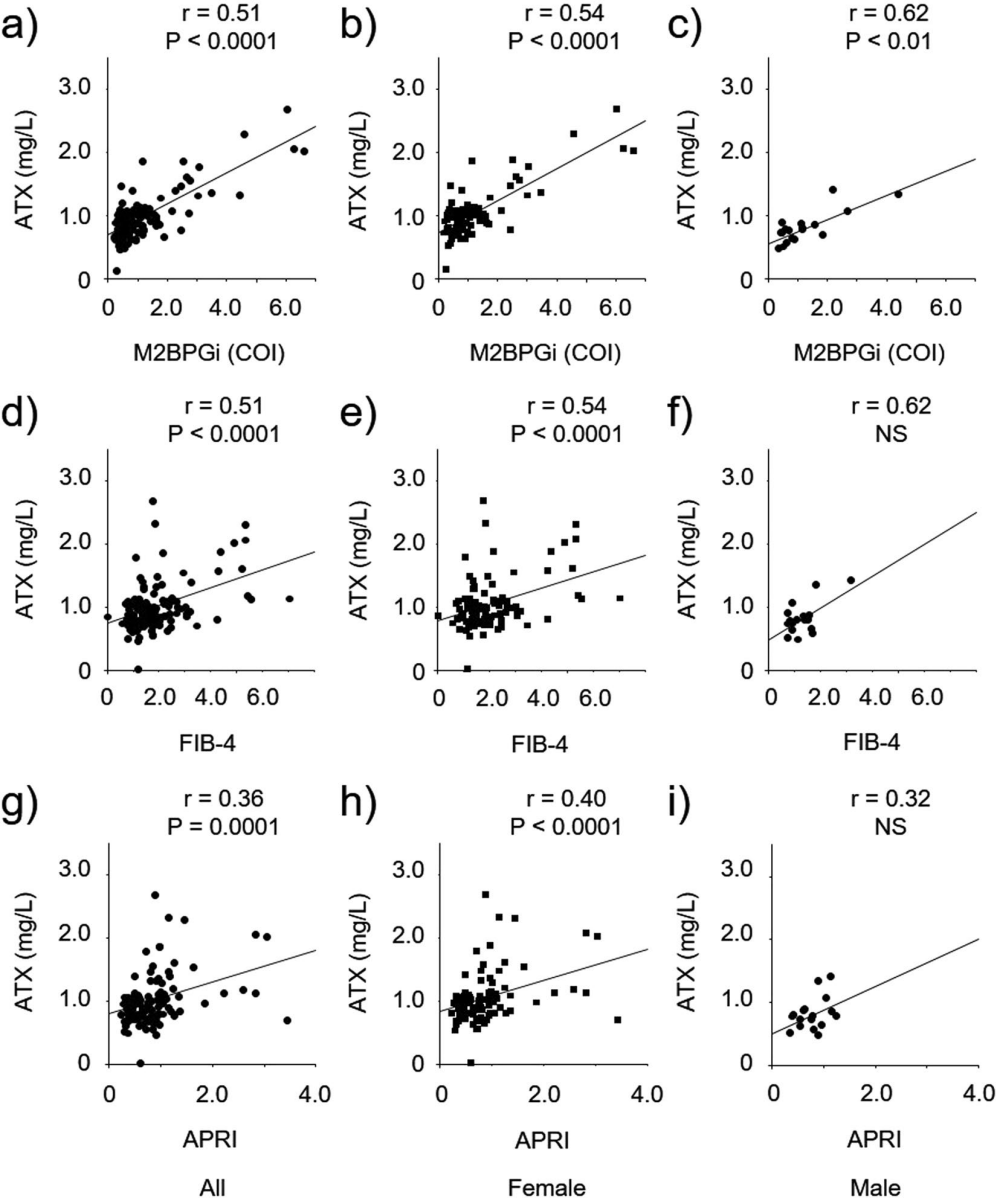
Correlation between ATX and other fibrosis markers, such as M2BPGi (**a**–**c**), FIB-4 (**d**–**f**), and APRI (**g**–**i**), in all, female, and male patients. Abbreviations: autotaxin, ATX; M2BPGi, *Wisteria floribunda* agglutinin-positive Mac-2-binding protein; FIB-4, fibrosis index based on four factors; APRI, aspartate aminotransferase-to-platelet ratio; NS, not significant.

The correlation coefficients between ATX and other clinical markers (ALP, GGTP, ALT, IgM, and NUP62) are listed in Supplementary Table 1. No significant associations between ATX and ALP, ALT, IgM, or NUP62 were found in overall patients, although weak significant correlations were detected in females. The correlations between ATX and the other tested non-invasive fibrosis markers (M2BPGi, FIB-4, and APRI) are presented in Fig. 2 and Supplementary Table 1. Moderate but statistically significant associations were observed between ATX and virtually all of the markers both overall and according to gender, apart from FIB-4 and APRI in males (Fig. 2a–i).

### Diagnostic ability of ATX and other fibrosis markers for predicting liver cirrhosis

In total, 18 patients were diagnosed as having Nakanuma’s liver cirrhosis stage 4, which included all 11 patients determined as Scheuer’s stage IV. We assessed the diagnostic ability of ATX to determine liver cirrhosis stage according to Nakanuma’s classification using receiver operating characteristic (ROC) analysis. As seen in Supplementary Figure 1 and Table 2, the ROC curves (AUROC) for ATX in diagnosing liver cirrhosis in overall, female, and male patients were all relatively high at 0.925, 0.968, and 0.984, respectively. The ATX results for AUROC, optimal cutoff value, sensitivity, specificity, positive predictive value, and negative predictive value in relation to liver cirrhosis stage are summarized in Table 2. ATX had the highest or near highest discrimination for liver cirrhosis stage, and significant differences were observed between the AUROC of ATX and those of FIB-4 index and APRI in female patients (P = 0.042 and P = 0.019, respectively).

**Table 2:** Diagnostic ability of ATX in assessing primary biliary cholangitis liver cirrhosis stage.

	Cutoff	AUROC	Sensitivity (%)	Specificity (%)	Positive predictive value (%)	Negative predictive value (%)
Overall	1.13	0.925	89	89	55	98
Female	1.19	0.968	85	89	52	98
Male	1.06	0.984	75	94	75	94

Abbreviations: ATX, autotaxin; AUROC, area under the receiver operating characteristic curve.

### Longitudinal study evaluating the clinical features of ATX

#### Clinical features of ATX in patients with PBC over 12 months of UDCA treatment

**Figure 3:**
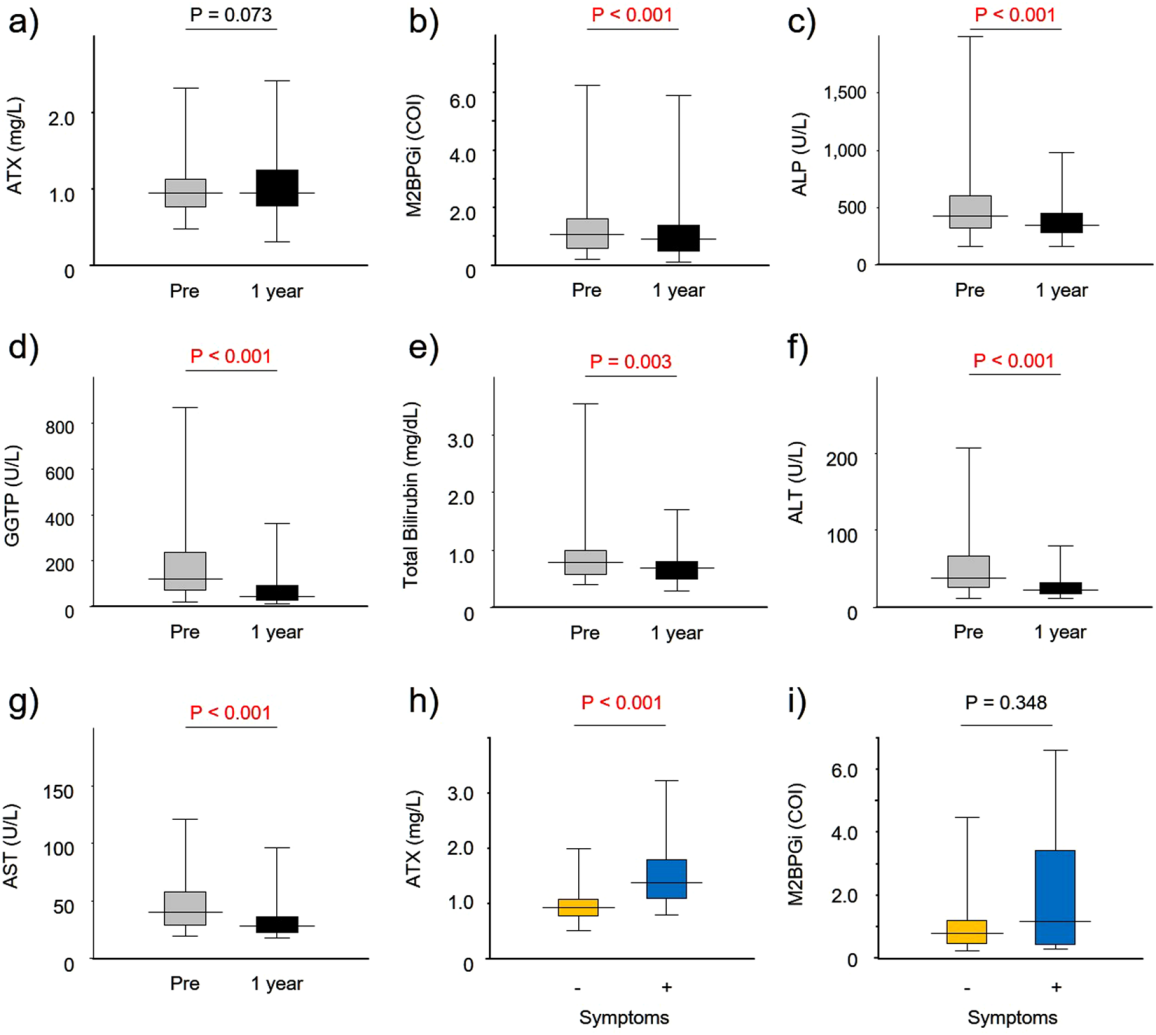
(**a**–**g**) Comparison of clinical parameters at diagnosis (Pre) and at 12 months of treatment with ursodeoxycholic acid (1 year). (**h**,**i**) Comparison of ATX and M2BPGi levels between asymptomatic and symptomatic patients at diagnosis. Abbreviations: ATX, autotaxin; M2BPGi, *Wisteria floribunda* agglutinin-positive Mac-2-binding protein; ALP, alkaline phosphatase; GGTP, gamma-glutamyl transpeptidase; ALT, alanine aminotransferase; AST, aspartate aminotransferase, −, asymptomatic patients; +, symptomatic patients.

All 128 patients with PBC in this study commenced oral UDCA therapy at a daily median dose of 600 mg/day (ranging from 300 mg/day to 900 mg/day). The percentage of patients who met the Paris criteria^18^, Barcelona criteria^44^, Toronto criteria^45^, and Ehime criteria^46^ were 91.3%, 47.6%, 84.6%, and 80.6%, respectively. Unlike M2BPGi, ALP, GGTP, T-bilirubin, AST, and ALT (Fig. 3b–g), ATX did not decrease remarkably (Fig. 3a) during the 12-month UDCA treatment course.

#### Clinical features of ATX in long-term follow-up patients with PBC

**Figure 4:**
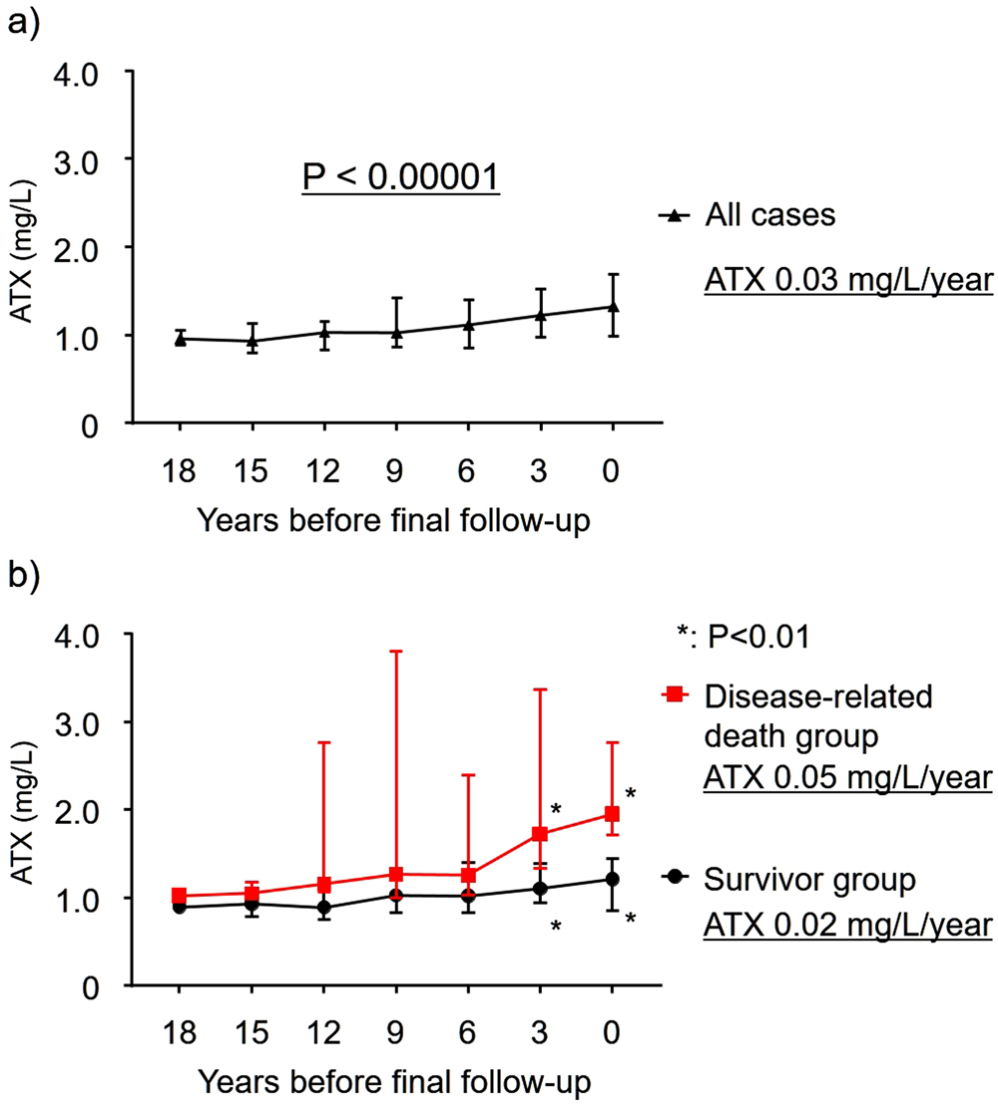
Clinical features of serum autotaxin (ATX) in long-term follow-up patients with primary biliary cholangitis. ATX levels were measured at the final follow-up and every 3 years beforehand in 29 patients. ATX increased significantly during follow-up in all patients (P < 0.00001). ATX levels in 21 survivors and 8 patients who succumbed to disease-related death were significantly different at 3 years before the final follow-up and at the final follow-up (P < 0.01).

The clinical characteristics of 29 patients (27 female and 2 male) in an independent second cohort, who had been seen regularly at our hospital for more than 18 years and had provided periodic serum samples, are summarized in Table 3. All patients were initially administered UDCA therapy. ATX levels were measured at the final follow-up and every 3 years prior in 21 survivors and 8 patients who eventually succumbed to disease-related death. There were no significant differences between the survivor and mortality groups for several clinical markers at PBC diagnosis. However, ATX slowly, but significantly increased during longitudinal follow-up, with a median increase rate of 0.03 mg/L/year (P < 0.00001) (Fig. 4a). At 18, 15, 12, 9, and 6 years before the final follow-up, there were no significant differences in ATX levels between survivors and disease-related death patients. However, ATX was significantly increased in the mortality group at 3 years before and at the final follow-up (P < 0.01). Longitudinal analysis of patient ATX values over 18 years revealed a significant difference in the ATX increase rates of survivors (0.02 mg/L/year) and patients with disease-related death (0.05 mg/L/year) (P < 0.01) (Fig. 4b).

**Table 3:** Clinical characteristics of 29 patients with primary biliary cholangitis in a longitudinal study of 18 years.

Baseline characteristic	Overall (n = 29)		Survivor (n = 21)		Death (n = 8)		Survivor vs. death
	Median	IQR	Median	IQR	Median	IQR	P value
Age (years)	55	(45–61)	55	(46–61)	56	(44–63)	0.83
Female/Male	27/2		19/2		8/0		0.37
**Laboratory data**							
alkaline phosphatase (U/L)	413	(314–635)	405	(314–635)	500	(310–644)	0.78
gamma-glutamyl transpeptidase (U/L)	97	(53–210)	100	(72–267)	46	(28–170)	0.10
alanine aminotransferase (U/L)	35	(30–52)	36	(30–52)	33	(29–55)	0.64
aspartate aminotransferase (U/L)	42	(32–54)	41	(35–54)	48	(31–58)	0.68
total bilirubin (mg/dL)	0.81	(0.60–0.81)	0.87	(0.60–0.94)	0.75	(0.59–1.06)	0.87
albumin (g/dL)	4.0	(3.8–4.3)	4.1	(3.9–4.3)	3.7	(3.5–4.4)	0.23
platelet count (x10^4^/µL)	19.1	(16.3–22.6)	18.8	(16.4–22.6)	19.4	(10.1–22.7)	0.74
immunoglobulin M (mg/dL)	312	(176–614)	355	(234–614)	180	(120–595)	0.20
immunoglobulin G (mg/dL)	1,576	(1,306–1,878)	1,576	(1,306–1,807)	1,816	(1,209–2,325)	0.45
AMA-M2 positive (%)	24 (83%)		18 (86%)		6 (75%)		0.49
anti-nuclear antibody positive (%) (x80)	23 (79%)		16 (71%)		8 (100%)		0.09
**Fibrosis markers**							
ATX (mg/L)	0.93	(0.86–1.07)	0.91	(0.79–1.29)	1.11	(1.02–1.17)	0.06
fibrosis index based on four factors index	1.91	(1.73–2.48)	1.86	(1.73–2.45)	2.41	(1.58–5.96)	0.29
aspartate aminotransferase-to-platelet ratio index	0.77	(0.61–1.03)	0.77	(0.68–1.00)	0.86	(0.50–2.98)	0.74
**Initial treatment**							
ursodeoxycholic acid 300/600/900	6/22/1		5/15/1		1/7/0		0.63

Data are expressed as the number (%) or median (first-third quartiles).

Abbreviations: IQR, interquartile range; AMA-M2, anti-mitochondrial antibody specific for the pyruvate dehydrogenase complex-E2 component; ATX, autotaxin.

## Discussion

This study demonstrated a clear association of ATX with disease progression in patients with PBC. Moreover, ATX results were validated by biopsy-proven histological assessment in all cases and were well correlated with other established non-invasive fibrosis markers, indicating ATX to be a reliable clinical surrogate marker to predict disease progression in patients with PBC. To validate the association of ATX with disease progression, we analyzed disease stage according to two classification systems^42,43^. ATX showed the highest resolution between non-cirrhotic stage and cirrhotic stage in these histologically proven classifications, which strongly supported a previous clinical report^41^. Indeed, another study described ATX as a prognostic factor for overall survival in patients with cirrhosis^37^, suggesting an important role of ATX in the progression of chronic liver diseases. As advanced histological stage is associated with a worse prognosis in PBC patients, it is important for clinicians to know clinical stage non-invasively when deciding appropriate therapies.

ATX values differed between female and male patients with PBC, which was consistent with earlier reports in healthy controls^36,47^ and in case studies^32,36,47,48^. Specifically, ATX levels were higher in females, which highlighted a need to establish gender-specific benchmarks. As the gender differences in PBC were similar to those reported in earlier viral infection studies on HCV^36^ and HBV^48^, clinicians should consider ATX-related gender differences regardless of etiology in the clinical setting. In fact, using ATX to assess PBC disease stage may prove to be advantageous since the majority of patients with PBC are female. Tokumura *et al*. observed increased serum ATX in normal pregnant women during the third trimester of pregnancy and elevated ATX in females with threatened preterm delivery^29^. Additional study is required to clarify the reason behind these gender discrepancies.

The prognosis of patients with PBC has improved greatly over the past two decades thanks to earlier diagnosis and the widespread use of UDCA. In support of this, most patients in our cohort were asymptomatic when diagnosed. UDCA therapy delays the progression to end-stage liver disease, enhances survival, and is well tolerated^18^. Patients treated with UDCA at a mild disease stage who achieve a biochemical response have a better prognosis than those with more advanced disease or who do not respond to the drug. In this regard, the extent of the biochemical response to UDCA during the first year of therapy is a simple and useful marker of long-term prognosis^18^. We examined the biochemical changes of several liver enzymes, including ALP and GGTP, and confirmed a significant biochemical response in patients at 12 months of UDCA therapy, although ATX levels did not decrease remarkably. Moreover, ATX slowly but significantly increased in longitudinal follow-up patients, which supported a previous study whereby ATX activity was higher in patients with a longer disease duration^41^. These findings may indicate that PBC-related fibrosis can slowly progress even with a biochemical response to UDCA. Obeticholic acid might be an alternative to UDCA but is not widely available, is unapproved in Japan, and has not been demonstrated to improve survival or disease-related symptoms^49^. Thus, other agents are needed to halt disease progression in PBC.

In terms of disease progression estimation markers, we compared ATX levels with autoantibodies such as gp-210^19^ and NUP62^50^ that have been reportedly associated with PBC disease progression. No statistically significant differences were found between ATX and these autoantibodies, specifically at the time of diagnosis. This result was supported in the longitudinal study whereby ATX did not differ at diagnosis, but was significantly increased in the mortality group at 3 years before and at the final follow-up (Fig. 4b). Therefore, clinicians may use ATX as a disease progression marker to estimate patient outcome together with established disease markers for better clinical decisions.

ATX is currently the only marker identified to correlate with the severity of cholestatic pruritus^49^. However, the vast majority of our patients showed no symptoms when diagnosed. We compared ATX levels at diagnosis in asymptomatic and symptomatic patients and observed that the latter group showed significantly higher ATX values (Fig. 3h) despite M2BPGi levels not differing between the groups (Fig. 3i). This indicates that ATX has pleiotropic functions in addition to its role in liver fibrosis; indeed, Nakamura *et al*. reported that serum levels of ATX became significantly decreased after radical prostatectomy^47^. It was recently described that ATX levels decreased significantly from baseline in a 10 mg obeticholic acid-treated group, although no correlation was observed between ATX activity and patient-reported measures of pruritus severity^49^. It was also revealed that ATX levels normalized or partially normalized under HCV treatment^51,52^ and that ATX was related to HCV infection and replication^53^. Our data showed moderate but significant associations of ATX with several laboratory parameters, including ALP, ALT, and IgM. These findings indicated that ATX levels were correlated with not only liver fibrosis, but also inflammation.

The present investigation has several limitations. It was retrospective and single-center in nature. The sample size was limited because we selected treatment-naïve patients to eliminate possible confounding factors on histology, such as a UDCA therapy. Liver biopsy for evaluating the degree of liver fibrosis was prone to sampling error. We also cannot exclude the possibility that ATX levels may have been underestimated since biopsy is sometimes contraindicated in cirrhotic stage patients due to a bleeding tendency or risk. A longer longitudinal investigation of patients with PBC with respect to ATX and clinical features, including long-term prognosis and complicating HCC, is warranted.

In conclusion, our findings show that serum ATX level represents an accurate, non-invasive biomarker for estimating disease progression in patients with PBC.

## Materials and Methods

### Patients and Methods

A total of 342 patients who were diagnosed as having PBC at Shinshu University Hospital between 1981 and 2016 were initially recruited for this study. As mentioned in several guidelines wherein histological assessment was not necessary to diagnose PBC^13–15^, 175 patients were diagnosed without histological assessment and/or sufficient serum or laboratory data and excluded. Thirty-nine patients had already received treatment with UDCA at the time of liver biopsy. Ultimately, 128 treatment-naïve patients with PBC were enrolled for a case-control study to identify associations of ATX with disease stage. The diagnosis of PBC was based on criteria from the Japan Society of Hepatology^15^. Serum AMA specific for the pyruvate dehydrogenase complex-E2 component (AMA-M2) was measured by the enzyme-linked immunosorbent assay, for which >7.0 U/mL was considered a positive result. No patient had a history of organ transplantation or concurrent use of immunomodulatory drugs or corticosteroids, and none were coinfected with the hepatitis C virus (HCV) or hepatitis B virus (HBV) or exhibited evidence of alcoholic liver disease or non-alcoholic fatty liver disease. The subjects were followed at regular intervals throughout the median observation period of 6.0 years (range: 1.0–33.0 years).

A separate additional 29 patients with PBC (8 eventually succumbing to disease-related death and 21 surviving) who had been treated with UDCA for at least 18 years and whose serum was cryogenically stored were recruited for a longitudinal study to uncover the clinical features of ATX over time during UDCA therapy.

One hundred and sixty subjects (80 male and 80 female) whose liver function tests were within normal levels were also enrolled as healthy controls^36^.

This investigation was reviewed and approved by the Institutional Review Board of Shinshu University School of Medicine (Matsumoto, Japan) (approval number: 3244). Written informed consent was obtained from all participating subjects. This study was conducted in accordance with the principles of the 1975 Declaration of Helsinki as revised in 1983.

### Detection of serum ATX

Blood samples were obtained and immediately stored at −20 °C until testing. Serum ATX antigen concentrations were simultaneously measured using cryogenically stored serum samples by a specific two-site enzyme immunoassay with an AIA-2000 system (Tosoh Co., Tokyo, Japan) as described previously^47^.

### Detection of gp210 and NUP62 autoantibodies

Serum antibody titers to gp210 were determined using an ELISA kit (INOVA Diagnostics, San Diego, CA, USA), whereby a value of ≥25 U was interpreted as a positive finding according to the manufacturer’s protocol and instructions. Serum antibody levels to NUP62 were also measured using an ELISA kit (LSBio, Seattle, WA, USA), in which detection levels ranged from 0.313 to 20 ng/mL according to the manufacturer.

### Fibrosis markers

The recently established M2BPGi fibrosis marker was quantified as described previously^17,24,25^. FIB-4 and APRI index were calculated as reported previously^28,54^.

### Histological evaluation

Liver biopsies were performed on all patients by percutaneous sampling of the right lobe with a 14-gauge needle as reported previously^55^. All liver biopsy samples were independently evaluated by two investigators who were blinded to the clinical results. Disease stage was determined according to Nakanuma’s classification^42^ and Scheuer’s classification^43^ systems.

### Statistical analysis

Statistical analysis and data visualization were carried out using StatFlex version 6.0 software (Artech Co., Ltd., Osaka, Japan). Data are presented as the median ± interquartile range for continuous variables. Groups were compared by means of the chi-square test for categorical variables. Correlations between fibrosis stage and serum ATX values were analyzed using Spearman’s rank test. Diagnostic accuracy was evaluated using the area under the AUROC. Cutoff values were identified by the Youden index, with the nearest clinically applicable value to the cutoff being considered as the optimal cutoff value for clinical convenience. All statistical tests were two-sided and evaluated at the 0.05 level of significance.

## Electronic supplementary material

Supplementary information

## References

[1] Kaplan MM, Gershwin ME (2005). Primary biliary cirrhosis. N Engl J Med.

[2] Kim WR (2000). Epidemiology and natural history of primary biliary cirrhosis in a US community. Gastroenterology.

[3] Joshita S (2010). Association analysis of cytotoxic T-lymphocyte antigen 4 gene polymorphisms with primary biliary cirrhosis in Japanese patients. Journal of hepatology.

[4] Umemura T (2016). Genetic Association of PTPN22 Polymorphisms with Autoimmune Hepatitis and Primary Biliary Cholangitis in Japan. Scientific reports.

[5] Hirschfield GM (2009). Primary biliary cirrhosis associated with HLA, IL12A, and IL12RB2 variants. The New England journal of medicine.

[6] Liu X (2010). Genome-wide meta-analyses identify three loci associated with primary biliary cirrhosis. Nature genetics.

[7] Mells GF (2011). Genome-wide association study identifies 12 new susceptibility loci for primary biliary cirrhosis. Nature genetics.

[8] Juran BD (2012). Immunochip analyses identify a novel risk locus for primary biliary cirrhosis at 13q14, multiple independent associations at four established risk loci and epistasis between 1p31 and 7q32 risk variants. Human molecular genetics.

[9] Nakamura M (2012). Genome-wide association study identifies TNFSF15 and POU2AF1 as susceptibility loci for primary biliary cirrhosis in the Japanese population. American journal of human genetics.

[10] Kawashima M (2017). Genome-wide association studies identify PRKCB as a novel genetic susceptibility locus for primary biliary cholangitis in the Japanese population. Human molecular genetics.

[11] Gershwin ME, Mackay IR (2008). The causes of primary biliary cirrhosis: Convenient and inconvenient truths. Hepatology.

[12] Van de Water J (1989). Detection of autoantibodies to recombinant mitochondrial proteins in patients with primary biliary cirrhosis. The New England journal of medicine.

[13] Lindor KD (2009). Primary biliary cirrhosis. Hepatology (Baltimore, Md.).

[14] EASL Clinical Practice Guidelines: The diagnosis and management of patients with primary biliary cholangitis. *Journal of hepatology* **67**, 145–172, 10.1016/j.jhep.2017.03.022 (2017). 10.1016/j.jhep.2017.03.022 28427765

[15] Guidelines for the management of primary biliary cirrhosis: The Intractable Hepatobiliary Disease Study Group supported by the Ministry of Health, Labour and Welfare of Japan. *Hepatology research: the official journal of the Japan Society of Hepatology* **44** Suppl S1, 71–90, 10.1111/hepr.12270 (2014). 10.1111/hepr.12270 24397841

[16] Joshita S, Umemura T, Ota M, Tanaka E (2014). AST/platelet ratio index associates with progression to hepatic failure and correlates with histological fibrosis stage in Japanese patients with primary biliary cirrhosis. Journal of hepatology.

[17] Umemura T (2015). Serum Wisteria floribunda Agglutinin-Positive Mac-2-Binding Protein Level Predicts Liver Fibrosis and Prognosis in Primary Biliary Cirrhosis. The American journal of gastroenterology.

[18] Corpechot C (2008). Biochemical response to ursodeoxycholic acid and long-term prognosis in primary biliary cirrhosis. Hepatology (Baltimore, Md.).

[19] Nakamura M (2007). Anti-gp210 and anti-centromere antibodies are different risk factors for the progression of primary biliary cirrhosis. Hepatology (Baltimore, Md.).

[20] Umemura T (2012). Human leukocyte antigen class II molecules confer both susceptibility and progression in Japanese patients with primary biliary cirrhosis. Hepatology (Baltimore, Md.).

[21] Yasunami M (2017). Principal contribution of HLA-DQ alleles, DQB1*06:04 and DQB1*03:01, to disease resistance against primary biliary cholangitis in a Japanese population. Scientific reports.

[22] Bravo AA, Sheth SG, Chopra S (2001). Liver biopsy. The New England journal of medicine.

[23] Castera L (2012). Noninvasive methods to assess liver disease in patients with hepatitis B or C. Gastroenterology.

[24] Ichikawa, Y. *et al*. Serum Wisteria floribunda agglutinin-positive human Mac-2 binding protein may predict liver fibrosis and progression to hepatocellular carcinoma in patients with chronic hepatitis B virus infection. *Hepatology research: the official journal of the Japan Society of Hepatology*, 10.1111/hepr.12712 (2016). 10.1111/hepr.12712 27029022

[25] Kuno A (2013). A serum “sweet-doughnut” protein facilitates fibrosis evaluation and therapy assessment in patients with viral hepatitis. Scientific reports.

[26] Toshima T (2015). A novel serum marker, glycosylated Wisteria floribunda agglutinin-positive Mac-2 binding protein (WFA(+)−M2BP), for assessing liver fibrosis. J Gastroenterol.

[27] Trivedi PJ (2014). Optimising risk stratification in primary biliary cirrhosis: AST/platelet ratio index predicts outcome independent of ursodeoxycholic acid response. Journal of hepatology.

[28] Sterling RK (2006). Development of a simple noninvasive index to predict significant fibrosis in patients with HIV/HCV coinfection. Hepatology (Baltimore, Md.).

[29] Tokumura A (2002). Identification of human plasma lysophospholipase D, a lysophosphatidic acid-producing enzyme, as autotaxin, a multifunctional phosphodiesterase. The Journal of biological chemistry.

[30] Umezu-Goto M (2002). Autotaxin has lysophospholipase D activity leading to tumor cell growth and motility by lysophosphatidic acid production. The Journal of cell biology.

[31] Stracke ML (1992). Identification, purification, and partial sequence analysis of autotaxin, a novel motility-stimulating protein. The Journal of biological chemistry.

[32] Nakagawa H (2011). Autotaxin as a novel serum marker of liver fibrosis. Clinica chimica acta; international journal of clinical chemistry.

[33] Ikeda H (1998). Effects of lysophosphatidic acid on proliferation of stellate cells and hepatocytes in culture. Biochemical and biophysical research communications.

[34] Yanase M (2000). Lysophosphatidic acid enhances collagen gel contraction by hepatic stellate cells: association with rho-kinase. Biochemical and biophysical research communications.

[35] Yanase M (2003). Functional diversity between Rho-kinase- and MLCK-mediated cytoskeletal actions in a myofibroblast-like hepatic stellate cell line. Biochemical and biophysical research communications.

[36] Yamazaki T (2017). Association of Serum Autotaxin Levels with Liver Fibrosis in Patients with Chronic Hepatitis C. Scientific reports.

[37] Pleli T (2014). Serum autotaxin is a parameter for the severity of liver cirrhosis and overall survival in patients with liver cirrhosis–a prospective cohort study. PloS one.

[38] Kondo M (2014). Increased serum autotaxin levels in hepatocellular carcinoma patients were caused by background liver fibrosis but not by carcinoma. Clinica chimica acta; international journal of clinical chemistry.

[39] Joshita, S. *et al*. Serum Autotaxin Is a Useful Liver Fibrosis Marker in Patients with Chronic Hepatitis B Virus Infection. *Hepatology research: the official journal of the Japan Society of Hepatology* (2017 in press). 10.1111/hepr.12997 29114991

[40] Kremer, A. E. *et al*. Lysophosphatidic acid is a potential mediator of cholestatic pruritus. *Gastroenterology* **139**, 1008–1018, 1018 e1001, 10.1053/j.gastro.2010.05.009 (2010). 10.1053/j.gastro.2010.05.009 20546739

[41] Wunsch E (2016). Serum Autotaxin is a Marker of the Severity of Liver Injury and Overall Survival in Patients with Cholestatic LiverDiseases. Scientific reports.

[42] Nakanuma Y (2010). Application of a new histological staging and grading system for primary biliary cirrhosis to liver biopsy specimens: Interobserver agreement. Pathology international.

[43] Scheuer P (1967). Primary biliary cirrhosis. Proceedings of the Royal Society of Medicine.

[44] Pares A, Caballeria L, Rodes J (2006). Excellent long-term survival in patients with primary biliary cirrhosis and biochemical response to ursodeoxycholic Acid. Gastroenterology.

[45] Kumagi T (2010). Baseline ductopenia and treatment response predict long-term histological progression in primary biliary cirrhosis. The American journal of gastroenterology.

[46] Azemoto N (2011). Biochemical response to ursodeoxycholic acid predicts long-term outcome in Japanese patients with primary biliary cirrhosis. Hepatology research: the official journal of the Japan Society of Hepatology.

[47] Nakamura K (2008). Validation of an autotaxin enzyme immunoassay in human serum samples and its application to hypoalbuminemia differentiation. Clinica chimica acta; international journal of clinical chemistry.

[48] Joshita S (2018). Serum autotaxin is a useful liver fibrosis marker in patients with chronic hepatitis B virus infection. Hepatology research: the official journal of the Japan Society of Hepatology.

[49] Nevens F (2016). A Placebo-Controlled Trial of Obeticholic Acid in Primary Biliary Cholangitis. The New England journal of medicine.

[50] Wesierska-Gadek J (2006). Correlation of initial autoantibody profile and clinical outcome in primary biliary cirrhosis. Hepatology (Baltimore, Md.).

[51] Kostadinova L (2016). During Hepatitis C Virus (HCV) Infection and HCV-HIV Coinfection, an Elevated Plasma Level of Autotaxin Is Associated With Lysophosphatidic Acid and Markers of Immune Activation That Normalize During Interferon-Free HCV Therapy. The Journal of infectious diseases.

[52] Yamazaki T (2018). Changes in serum levels of autotaxin with direct-acting antiviral therapy in patients with chronic hepatitis C. PloS one.

[53] Farquhar MJ (2017). Autotaxin-lysophosphatidic acid receptor signalling regulates hepatitis C virus replication. Journal of hepatology.

[54] Wai CT (2003). A simple noninvasive index can predict both significant fibrosis and cirrhosis in patients with chronic hepatitis C. Hepatology (Baltimore, Md.).

[55] Umemura T (2007). Immunoglobin G4-hepatopathy: association of immunoglobin G4-bearing plasma cells in liver with autoimmune pancreatitis. Hepatology (Baltimore, Md.).

