# Supplementary material for: Serum Autotaxin Is a Useful Disease Progression Marker in Patients with Primary Biliary Cholangitis
Source: Sci Rep. 2018 May 25;8:8159. doi: 10.1038/s41598-018-26531-0 (PMC5970155; doi:10.1038/s41598-018-26531-0)

Serum Autotaxin Is a Useful Disease Progression Marker in Patients with Primary Biliary Cholangitis

Satoru Joshita, Takeji Umemura, Yoko Usami, Yuki Yamashita, Gary L. Norman, Ayumi Sugiura, Tomoo Yamazaki, Naoyuki Fujimori, Takefumi Kimura, Akihiro Matsumoto, Koji Igarashi, Kaname Yoshizawa, Masao Ota, Eiji Tanaka

Table of contents

Supplementary Table 1……………………………………………………………….2

Supplementary Figure 1………………………………………………………………3

**Supplementary Table 1. Correlation coefficients between autotaxin and other clinical and fibrosis markers**

|  |  | ALP | GGTP | ALT | IgM | NUP62 | M2BPGi | FIB-4 index | APRI |
| --- | --- | --- | --- | --- | --- | --- | --- | --- | --- |
| Overall | r | 0.18 | 0.02 | 0.08 | 0.10 | 0.00 | 0.51 | 0.37 | 0.36 |
|  | P | NS | NS | NS | NS | NS | <0.0001 | <0.0001 | 0.0001 |
| Female | r | 0.30 | 0.15 | 0.20 | 0.20 | 0.01 | 0.54 | 0.30 | 0.40 |
|  | P | 0.002 | NS | 0.039 | 0.040 | NS | <0.0001 | 0.002 | <0.0001 |
| Male | r | -0.31 | -0.19 | -0.05 | -0.41 | -0.04 | 0.62 | 0.28 | 0.32 |
|  | P | NS | NS | NS | NS | NS | <0.01 | NS | NS |

Abbreviations: ALP, alkaline phosphatase; GGTP, gamma-glutamyl transpeptidase; ALT, **alanine aminotransferase;** IgM, immunoglobulin M; NUP62, nuclear pore glycoprotein p62; M2BPGi, *Wisteria floribunda* agglutinin-positive Mac-2-binding protein; FIB-4, fibrosis index based on four factors; APRI, aspartate aminotransferase-to-platelet ratio; NS, not significant.

Data were analyzed by Spearman’s rank correlation coefficient test for each gender.

Supplementary Figure 1.

Diagnostic ability of serum autotaxin (ATX) levels to assess disease progression in patients with primary biliary cholangitis. Receiver operating characteristic curves for ATX for the estimation of cirrhosis based on Nakanuma’s classification in all, female, and male patients.


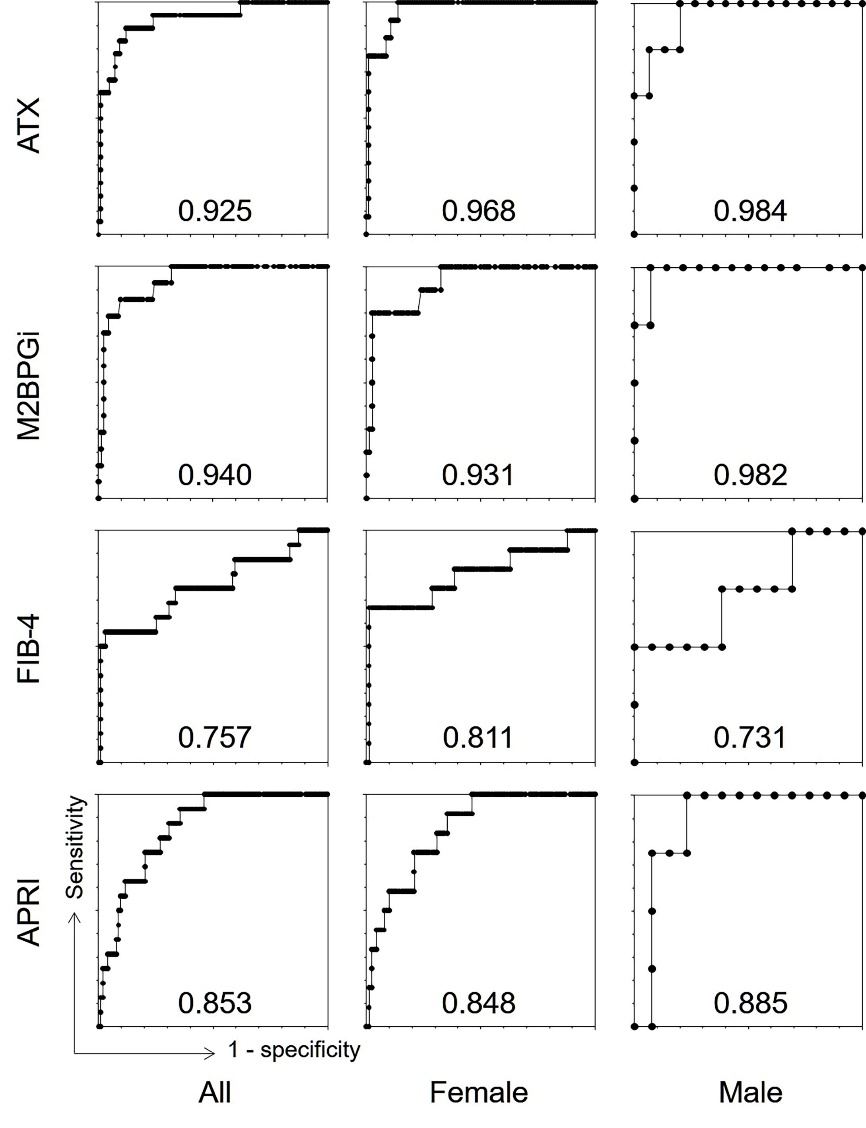

Supplement: Supplementary file 1 — Supplementary information [file 41598_2018_26531_MOESM1_ESM.docx]
